# An empirical study on asymmetric jump diffusion for option and annuity pricing

**DOI:** 10.1371/journal.pone.0216529

**Published:** 2019-05-07

**Authors:** Kein Joe Lau, Yong Kheng Goh, An Chow Lai

**Affiliations:** 1 Centre for Matheamtical Sciences, Universiti Tunku Abdul Rahman, Bandar Sungai Long, Kajang, Selangor, Malaysia; 2 Department of Electrical and Electronic Engineering, Universiti Tunku Abdul Rahman, Bandar Sungai Long, Kajang, Selangor, Malaysia; The Bucharest University of Economic Studies, ROMANIA

## Abstract

In this paper, we present a method to estimate the market parameters modelled by an asymmetric jump diffusion process. The method proposed is based on Kou’s jump diffusion model while the market parameters refer to the market drift, the market volatility, the jump intensity on market price, and the rate of jump occurrence in a consistent manner throughout the entire paper. The model captures the asymmetric nature of the price fluctuation during up trend markets and down trend markets. The results are compared to conventional options to observe the impact of jump effects. The results from simulation show that the asymmetric jump diffusion model can estimate the fair prices of European call options and annuity better than the Black-Scholes model and the symmetric jump diffusion model proposed by Kou and Merton.

## Introduction

It is well known that the financial market is volatile and difficult to predict. Nevertheless, investors are still trying to learn and forecast the financial market. The most commonly used method in modelling the stock price movement is the Brownian motion model. An example that applies the Brownian motion model in pricing option is the Black-Scholes model [1–3].

In the early 90s, the Black-Scholes model [4] was regarded to be one of the most favourable methods in calculating option prices. The Black-Scholes model was derived from the expected volatility (implied volatility) to project future prices of the financial assets while many investors use it to calculate the fair price of an option. During the 1997 financial crisis, most investors, including the experienced market investors, suffered from significant losses due to the drastic downward “jumps” in prices. This incidence had shown that Black-Sholes model might be useful to a certain extent, but it could not handle the extreme events where market movements are out of ordinary [5].

Much research had proposed modifications to both the Black-Scholes model and the jump diffusion model. In this paper, we will focus on the extension of the jump diffusion model, introduced by Kou [6] and Merton [7], which allows separate treatments for the up trend and down trend price movements. Carr et al. [8] had also studied the model with the assumption that infinite jump events are possible.

Bates [9] proposed an approach that used a stochastic volatility model along with the jump diffusion models. Bates’s model considers jumps that are in the value of an underlying asset. More extensive reviews on the jump diffusion model with a stochastic volatility model can refer to Eraker et al. [10].

Salmi and Toivanen [11] proposed an iterative method for pricing American options under the jump diffusion model. They compared the prices of both European option and American option with other researchers, i.e. D’Halluin [12] and Toivanen [13]. Their approaches provided similar results under predefined model parameters; i.e. when the jump diffusion models being used are identical. Chiarella and Ziogas added another method for pricing American option that applies the Fourier-Hermite series expansion [14].

Sidorov [15] used the GARCH model to show that the expected volatility of underlying asset changes over time. However, the changes are only significant if the measured period is long enough. In this paper, we investigate each period through a yearly basis; hence, the changes in volatility would be insignificant unless a jump event occurs.

Sidorov [16] also proposed several GARCH models to forecast two indices. He compared and investigated the forecasting ability of GARCH models using some standard performance measurements. Among all the models used, the better model is the EGARCH skewed Student-*t* model. However, the research could not show significant results to draw a general conclusion.

Dutta [17] studied the daily exchange rate of US Dollar to Japanese Yen by modelling the volatility using the symmetric and asymmetric GARCH models. The author assumed that the exchange rate changes followed the normal or a heavy-tailed distribution. The author’s analysis reveals that when the heavy-tailed distribution is considered, the persistence of the jumps is found to be reduced in each of the cases under studied. These few papers had shown that the GARCH model does not consider the scenario where jump events occurred in the underlying asset.

Here we propose an asymmetric jump diffusion model aimed to extend the usage of the Black-Scholes model with asymmetric jumps. Incorporating jumps into the Black-Scholes model is not new; most of the researchers had proposed different approaches that used the Kou or Merton jump diffusion model. Both models proposed by Merton and Kou assume symmetric jump distributions in both upward and downward directions, while through our study, we realised that a jump event could occur in both symmetric and asymmetric. Hence, in our model, we will treat the jumps in two directions separately.

The implication of this extension is that practitioners should treat the effects of the jumps in the upward and downward trends differently. The fact that market returns have asymmetric jumps is mostly well known in the stock market [18] and affects other derivatives. The study on asymmetric jumps is especially crucial for derivatives with different risks in the up trend markets or down trend markets [19]. Our empirical model that provides a way to estimate the jump intensities and demonstrates that the asymmetric property of the jump could affect the pricing of the derivatives. This information allows practitioners to have a clearer picture of the actual risk that they are taking as compared to the pricing model assumming symmetric volatility.

The outline of this paper is as follows. Next section provides a brief explanation of the geometric Brownian motion model and the jump diffusion model proposed by Kou and Merton. In the research methodology section, we will show our modification to the jump diffusion model, along with a brief introduction of the Gibbs sampling method, and the details of the empirical method that we use. Subsequently, we present the pricing models for European call and annuity products that use the Black-Scholes model and the Heston model respectively. This is followed by the results and data analysis of the empirical method and the asymmetric jump diffusion model. Last section is the discussion and conclusion. The main contributions of this paper are the empirical method and the modification of jump diffusion model proposed in the methodology.

### Geometric Brownian motion model and jump diffusion model

The geometric Brownian motion model is the basis of the Black-Scholes model. Mentioned by Samuelson [20], the stochastic process of the prices of an asset could be described as a geometric Brownian motion (GBM) in a form of a stochastic differential equation (SDE):

dS(t)=μS(t)dt+σS(t)dW(t), (1)

where *μ* is the drift rate or the rate of return; *σ* is the volatility of the asset; *W*(*t*) is a Wiener process, and *S*(*t*) is the spot price of the underlying assets.

A study by Adeosun [21] explains that anticipating a market takes much more than a geometric Brownian motion (GBM) which is considering only the volatility and drift of an asset. The pricing model solely based on GBM will not be sufficient to accommodate the market prices.

In the past 40 years, various models had been proposed to reflect the discontinuity and the jump in asset returns. These include Merton [7], Press [22], Bates [9], and Kou [6]. In these models, typically a compound Poisson component is added into the Black-Sholes model to emulate the jump component of the asset returns:

$$S\left(t\right)=S\left(0\right)e^{\left(\mu -\frac{1}{2}\sigma^{2}\right)+\sigma W\left(t\right)}\prod_{i=1}^{N(t)}e^{Y_{i}},$$ (2)

where *N*(*t*) is a Poisson process; *Y*_*i*_ is a standard normal distributed with the mean of zero and standard deviation of one; *μ* is the drift coefficient of underlying GBM while *σ* is the volatility of the underlying GBM.

Assuming the market follows a geometric Brownian motion, the additional Poisson process describes the arrival of jump events. The jump event has its drift and volatility terms that differ from those of the underlying GBM. Kou modified the above model, with *Y*_*i*_ being changed to the double exponential distribution [6]. Kou claimed that this modification would enable one to obtain analytical solutions for most path-dependent options, including barrier options and analytical approximations for American options [6]. The following expression describes Kou’s double exponential distribution for *Y*_*i*_:

$$f_{Y}\left(y\right)=p\cdot \eta_{1}\exp^{-\eta_{1}y}1_{\left\{y\geq 0\right\}}+q\cdot \eta_{2}\exp^{\eta_{2}y}1_{\left\{y<0\right\}},$$ (3)

where

- *η*_1_ > 1, *η*_2_ > 0;
- *p, q* ≥ 0, *p* + *q* = 1, representing the upward and downward jumps;
- *η*_1_ > 1 is required so that **E**(*e*^*y*^)<∞;
- **E**(*S*(*t*)) < ∞.

Kou had pointed out two properties of double exponential distribution are of importance for the model. The first one is the leptokurtic or “fat tail” feature of the jump size where it inherits the return distribution. This property makes sense as a jump of an instrument is not entirely random but depends on the characteristic of the instrument itself. For example, a stock price would not have a drastic hike that is several folds of magnitude difference relative to its spot price [23].

The second crucial property of the double exponential distribution is its martingale property. This unique property makes closed-form solutions (or approximations) for option pricing problems feasible. Besides, Kou’s model also has the advantage of being internally self-consistent and free of arbitrage in an equilibrium setting. The model can capture the empirical aspect of the stock markets, and the model parameters are straightforward to calibrate [24].

Kou shows that the jump diffusion model can improve the empirical implications of the Black-Scholes model, as it remains and retains its analytical tractability [25]. On the other hand, Chan and Wong had shown that the jump model could be used to attain better market pricing than the Black-Scholes model option pricing method using the geometric Brownian motion [26].

Therefore, this research aims to incorporate the jump diffusion model into the Black-Scholes model and show its applicability on European call options and annuity. The next section discusses how the jump model’s parameter is calculated as well as the impact of these parameters on option pricing.

## Research methodology

### Data collection

In this research, we chose the Dow Jones industrial (DJI), NASDAQ Composite 100 (NASDAQ 100), Financial Times Stock Exchange 100 Index(FTSE 100), Standard & Poor’s 500 (S&P 500) and NYSE ARCA oil & gas Index (OilGas). We retrieved those market data from Yahoo Finance covering the period from January 2005 to December 2014.

We separated the retrieved data into two different periods: (a) between 2005 to 2010, and (b) between 2011 to 2015. The first period covers the year 2007 and 2008, which is known as the economic crisis period. The second period is used as the control period. By doing this, we could compare the parameters for both sets of data.

### Gibbs sampling method and jump diffusion model parameters

In this research, we will use the Gibbs sampling method to obtain the values of the parameters *μ*, *σ*, *μ*_*jump*_ and λ_*jump*_ in the jump diffusion model [26, 27]. The fundamental idea of the Gibbs sampling method is using the Bayesian inference from Markov Chain Monte Carlo (MCMC) with Metropolis-Hasting model. Metropolis-Hasting model is an iterating process that updates initialised values towards the distribution with an accept-reject method while the Gibbs sampling technique is a particular case where we accept every update for the iterations [28, 29].

The model begins with a probability mass function (pmf), *π* on a countable set of states, *X* and a real-valued function, *f*(*X*). Here, both *π* and *f*(*X*) are assumed to be complicated and computing their exact values is intractable and exact sampling is impossible. Hence, we use the model to sample from *π* approximately, or to approximate the expected value **E**[*f*(*X*)] where *X* ∼ *π* and **E**[*f*(*X*)] is distributed according to *π*.

The steps of the Gibbs sampling algorithm are as follows:

1. We introduce the proposal matrix **Q**. **Q** is a stochastic matrix, where all its element is positive, and sum of each row is equal to 1. (*Q*_*ab*_ = *Q*_*ba*_ ∀*a*, *b* ∈ *X*)
2. Initialize **X**_0_ ∈ **X**. Where **X**_0_ is an element from **X**.
3. For iteration where *i* = 0, 1, 2, …, *n*-1:
  - Sample *x* from **Q**(*x*_*i*_, *x*), such that *x*_*i*_ is a fixed, known variable and *x* is the sample that range over all possible state, (or can be say as **P**(*x*∣*x*_*i*_) = *Q*(*x*_*i*_, *x*)).
  - Sample a *u* from uniform(0,1), where *u* represent the constant for the rate to accept or not accept in the next step.
  - If $u<\frac{\tilde{\pi }\left(x\right)}{\tilde{\pi }\left(x_{i}\right)}$ (the probability of *x*), then *x*_*i*+1_ = *x*, else we reject the newly drawn sample and *x*_*i*+1_ = *x*_*i*_.
4. The output is a sequence of *x*_0_, *x*_1_, *x*_2_, …, *x*_*n*_ as *i* goes from 1 to *n* − 1.

The iteration process will be used in sampling the market parameters. By iterating each different market parameter on their own posterior distributions, we can sample out the market parameters with past market prices.

We implemented a Gibbs sampler to sample out the parameters needed for Merton’s jump diffusion model; i.e. *μ*—the drift, *σ*—the volatility of the underlying asset, λ—the frequency of the jump event, *μ*_jump_—the intensity of the jumps, and *σ*_jump_—the volatility of the jumps. These parameters will show the behaviour and movement of the underlying asset; hence knowing these characteristics will provide insight for investors when managing risks.

Take note that there are two sources of fluctuations in the underlying asset prices. The term intensity of the jumps is used to make the distinction of the sudden up-surge or down-surge in the fluctuations of the underlying asset from the less drastic Gaussian fluctuations of the asset, i.e. the volatility.

In the context of parameter sampling with the Gibbs sampler, the proposal matrix **Q** would be the posterior distribution of parameters. We will let the initial parameter be *X*_0_ and will repeat the sampling process until it converges. The list of output will be the samples of the parameters.

The Table 1 reports the values of the parameters from the Gibbs Sampling method generated by a simulated jump diffusion model.

**Table 1:** Testing Gibbs sampler algorithm.

Parameters	Preset	Mean	Standard Deviation
Drift of Asset	0.1	0.00198	0.43901
Volatility of Asset	0.5	0.04132	0.02244
Frequency of Jump	10.0	9.51044	3.77489
Intensity of Jump	0.05	0.05295	0.08975
Volatility of Jump	0.025	0.07128	0.03999

The results show the Gibbs sampler’s limitations in converging for the parameter jump drift and standard deviation of the jump; i.e. the calculated mean of each simulated parameter deviates from the preset value while the standard deviation is higher than 30%. These show that the accuracy of the Gibbs sampling is not good enough to recover the preset parameters of the model.

### The empirical method

The values of the parameters are obtained by using an empirical method as the Gibbs sampling method is not good at distinguishing fluctuations from assets volatility or jump volatility; hence, unable to retrieve the modelling parameters effectively.

First, we calculate the daily rate of difference in close prices with respect to previous day for each day,

$${\text{diff}}_{i}={\text{price}}_{i}-{\text{price}}_{i-1},$$

where *i* goes from day 1 to day *n*. Next, we group the calculated differences according to their signs and find the medians for both positive and negative rate of differences separately, i.e. median^+^ and median^−^.

We redefine those differences that are four times larger than the positive median (or four times smaller than negative median) as the jumps. Hence, the average jump intesity for upward and downward, denoted as *Y*_1,*i*_ and *Y*_2,*i*_ respectively, are described in Eq 4. Our asymmetric model requries the jump intensity as *E*(jump)≥∣4 × *median*∣. This definition is different from the previous symmetric jump model that defines jump intensity at zero, *E*(jump) = 0.

The number of times of such jump values are treated as the frequency of jumps and will be used in the Poisson distribution of the jump event, *N*_1_(*t*) (or *N*_2_(*t*) for downward jump). Lastly, by taking the average log of the rate of return of the underlying asset, we would get the drift and volatility of the underlying asset, i.e. *μ* and *σ*.

**Fig 1:**
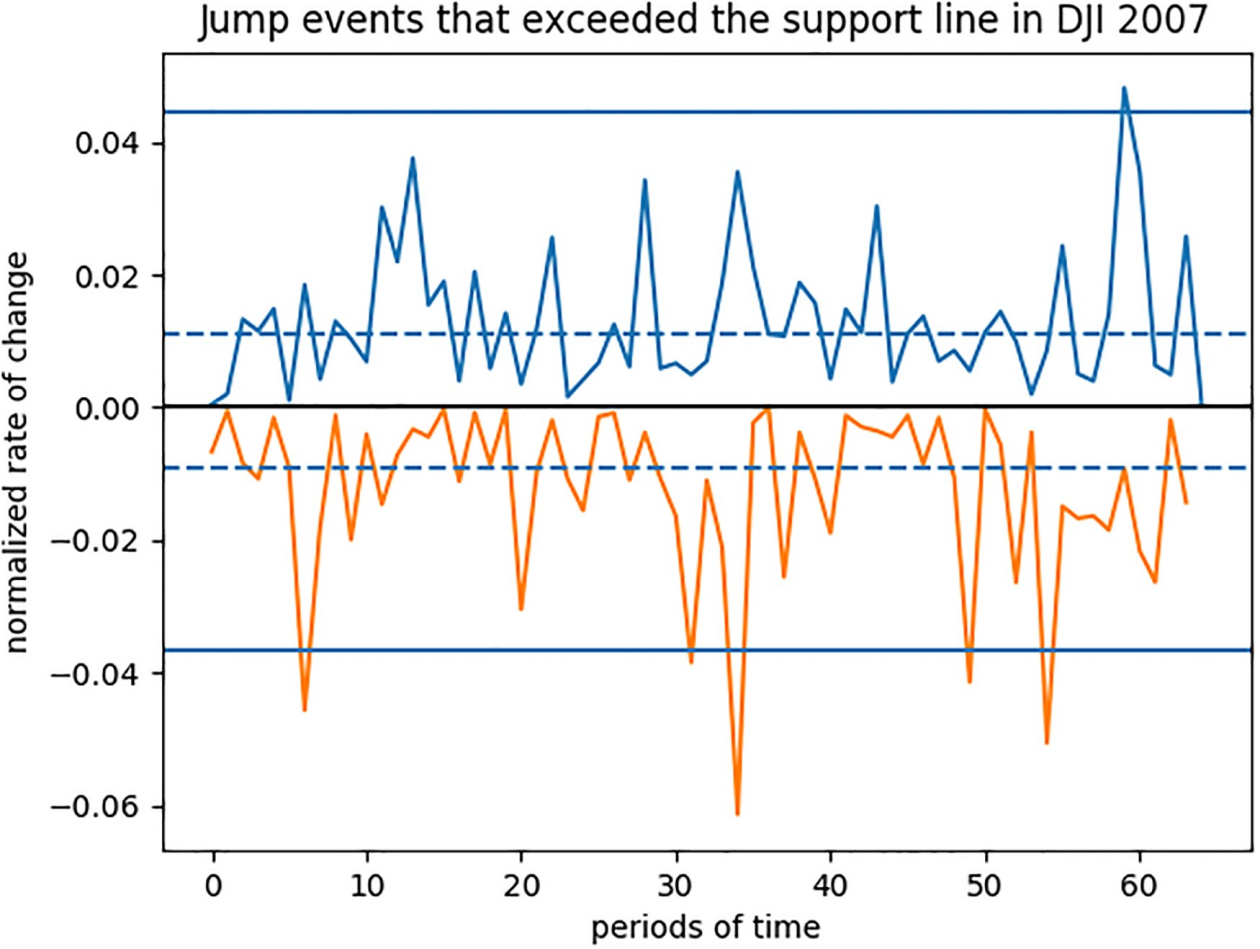
An illustration of jump event identification where the jumps exceeded the lines are considered as jump events.

For example, we use the data from Dow Jones in the year 2007 as illustrated in Fig 1. The *x*-axis is the period number and *y*-axis is the rate of changes in price. We will get 1 upward jump and 5 downward jumps, with average intensities of 0.012 and −0.0107 respectively. The drift and volatility of DJI at 2007 are −0.0043 and 0.1454 respectively.

The Table 2 is retrieved using the empirical methods on S&P 500 and DJI indices from the year 1995 until 2014.

**Table 2:** Jump parameters for DJI and S&P 500 from year 1995 to 2014.

DJI					S&P				
Year	Up	Up intensity	Down	Down intensity	Year	Up	Up intensity	Down	Down intensity
1995	0	0.000000	4	0.006452	1995	1	0.003583	5	0.007322
1996	5	0.006385	9	0.010148	1996	2	0.009151	11	0.008307
1997	0	0.000000	1	0.029571	1997	1	0.006960	1	0.025787
1998	3	0.010684	3	0.025415	1998	2	0.013205	8	0.017721
1999	1	0.009298	5	0.012175	1999	0	0.000000	1	0.013725
2000	1	0.040104	3	0.016139	2000	5	0.015921	2	0.018840
2001	4	0.016356	5	0.020903	2001	1	0.020283	3	0.021277
2002	5	0.028068	2	0.018651	2002	3	0.024552	2	0.030803
2003	1	0.015568	2	0.015361	2003	1	0.014023	2	0.014982
2004	2	0.007285	5	0.008304	2004	1	0.007017	3	0.010429
2005	1	0.008557	2	0.007423	2005	0	0.000000	2	0.009941
2006	0	0.000000	5	0.009115	2006	0	0.000000	5	0.008411
2007	1	0.012031	5	0.010785	2007	2	0.013596	3	0.012453
2008	3	0.052077	4	0.039357	2008	4	0.052090	7	0.048799
2009	2	0.017149	3	0.017408	2009	1	0.027948	5	0.022862
2010	1	0.009696	7	0.012784	2010	1	0.011456	10	0.013855
2011	2	0.023121	3	0.017075	2011	2	0.024247	4	0.016401
2012	3	0.007883	6	0.007448	2012	0	0.000000	4	0.010550
2013	3	0.005309	5	0.008180	2013	1	0.005189	3	0.011769
2014	4	0.008574	9	0.008321	2014	4	0.008849	5	0.013490

Up represents the frequency of upward jumps for the year, while down represents the frequency of downward jumps for the year.

From Table 2, the upward jumps are not in line with the downward jumps. The frequency of downward jumps is higher as compared to that of upward jumps. The intensity of jumps is different for each direction of the jump as well. Hence, we can conclude that it is essential to modify the model by Kou and Merton, to accommodate the two different directions of the jump.

### Modified double exponential jump diffusion models

As shown in Eq 3, the usage of the double exponential requires more parameters for Kou’s model. Besides the drift and volatility of the assets, *μ* and *σ*, Kou’s model requires *η*_1_ and *η*_2_ which are the mean intensity of upward and downward jumps for the double exponential distribution. Note that the frequency of jump, λ_jump_, in Kou’s model includes both upward and downward jump events.

Throughout the research, the results show that the intensity on upward jumps and downward jumps are independent of each other. Hence, we propose a method that splits the frequency of jump arrival and intensity of jumps into two different parameters each. This will ensure the retractability of upward and downward jumps, as each direction has its own frequency of jump and jump intensity. The results are as shown in Eq 4.

$$S\left(t\right)=S\left(0\right)e^{\left(\mu -\frac{1}{2}\sigma^{2}\right)+\sigma W\left(t\right)}\prod_{i=1}^{N_{1}\left(t\right)}e^{Y_{1,i}}\prod_{i=1}^{N_{2}\left(t\right)}e^{Y_{2,i}},$$ (4)

where *N*_1_(*t*) is a Poisson process for upward jump frequency in a period; *N*_2_(*t*) is a Poisson process for downward jump frequency in a period; *Y*_1,*i*_ is a standard normal distributed random variable for upward jump; *Y*_2,*i*_ is a standard normal distributed random variable for downward jump; *μ* is the drift coefficient for underlying asset; and *σ* is volatility of the underlying asset.

### Pricing European call with asymmetric jump diffusion model

The number of jump events is different for each market instrument. Generally it is believed that a market crisis will occur once every ten years. However, a jump does not always cause a drop in prices; an upward jump is also possible. How the investors should group and identify the jump aside from normal daily fluctuation will be a critical element to consider.

For the European call option price, we use the Black-Scholes model that was worked out and developed by three economists—Fisher Black, Myron Scholes and Robert Merton.

$$\begin{array}{c} C=S\;N\left(d_{1}\right)-K\exp^{\left(-rt\right)}\;N\left(d_{2}\right), \end{array}$$ (5)

where, *C* is the call premium at time zero, *S* is the current stock price, *K* is the strike price, *t* is the time to expiry, *r* is the risk-free rate, *σ* is volatility of the underlying asset, *N*(⋅) denotes the standard cumulative normal distribution, while $d_{1}=\frac{\ln\frac{S}{K}+\left(r+\frac{\sigma^{2}}{2}\right)t}{\sigma \sqrt{t}}$, and $d_{2}=d_{1}-\sigma \sqrt{t}$.

The stock price, *S* is calculated using the asymmetric jump diffusion model, in Eq 4. The results we achieved in Table 2 will be used in Eq 4. Hence, the option price will change according to the impact of the jump for the period.

Since the values of the jump parameters are different for different instruments, we set the value range of 0 to 4 for the frequency of jump and 0 to 0.08 for the intensity of jump (0 < λ <4, 0 <*μ*_jump_ <0.08). These ranges are selected, as per our research of 20-year data across 5 indices, where most of the jump frequency and intensity fall within these ranges. Note that the zero jump frequency is equivalent to a standard geometric Brownian motion model.

The initial price and strike price are set at $100. The drift, *μ* and volatility of the asset, *σ* values are fixed at 0.08 and 0.4 respectively, as calculated over 20 years of Dow Jones index for its average drift and volatility. We calculate the expected price of the European call option and results are shown in the result section.

### Pricing annuity with asymmetric jump diffusion model

The values of annuity vary depending on different requested requirements by the annuitant. A larger stream of income and insured guarantee in the future would cost higher premium (or a regular annuity payment) and vice versa. Under the Black-Scholes model assumptions, the dynamics of annuity account value are as follows [30]:

$$\begin{array}{c} dA_{t}=\left(\mu -c\right)A_{t}dt+\sigma A_{t}dB_{t}+kdt. \end{array}$$ (6)

where *A*_*t*_ and *c* are denoted as the sub account value at time *t* and the mortality and expense fee payable continuously respectively. While *k* is the subsequent contributions to the sub-account [31].

Under the Black-Scholes model, the combined GBM with jump event (Eq 2) model is given by the following SDE:

$$\begin{array}{c} dS_{t}=\mu S_{t}dt+\sigma S_{t}dB_{t}+JS_{t}dN_{t} \end{array}$$ (7)

In the case of an extreme event, the sub account value will be modified with jump diffusion model assumption and becomes:

$$\begin{array}{c} \begin{array}{cc} dA_{t} & =A_{t}\left(dS_{t}\right)/S_{t}-cA_{t}dt+kdt\\ & =A_{t}\left(dt+dB_{t}+JdN_{t}\right)-cA_{t}dt+kdt\\ & =\left(-c\right)A_{t}dt+A_{t}dB_{t}+JA_{t}dN_{t}+kdt \end{array} \end{array}$$ (8)

We consider annuitant has guarantee benefits with roll up premium, consisting of both guarantee minimum death benefits (GMDB) and guarantee minimum accumulation benefits (GMAB) [32]. The guarantee benefits had a pre-agreed guaranteed interest rate *g* ≥ 0, which is chosen such that *g* < *r*. Hence the guarantee benefits are given as follows:

$$\begin{array}{c} G_{t}=\{\begin{array}{c} A_{t}\left(dS_{t}\right)/S_{t}-cA_{t}dt+kdt\\ A_{t}\left(\mu dt+\sigma dB_{t}+JdN_{t}\right)-cA_{t}dt+kdt, \end{array} \end{array}$$ (9)

This guarantee benefits resemble an Asian put option where the sub-account value, *A*_*t*_ is treated as the underlying asset. The payoff function, *P*(*t*) will be given as follows:

$$\begin{array}{c} P\left(t\right)={\left[G\left(t\right)-A_{t}\right]}_{+}=\max\left(G_{t}-A_{t},0\right),\;\text{for}\;t\leq T \end{array}$$ (10)

The price of the annuity depends on the sub-account value, *A*_*t*_. This sub-account value price will fluctuate according to the market movement, similar to the stock price in the European call option. Hence, by changing the model of the account value from the GBM model to the asymmetric jump diffusion model, we can calculate and simulate the price of the annuity.

## Results and data analysis

### Gibbs sampler for market indices

From the Gibbs sampler we have retrieved the drift, standard deviation along with jump parameters in jump model described by Eq 2 for various market indices. We picked Dow Jones industrial (DJI), NASDAQ Composite 100 (NASDAQ 100), Financial Times Stock Exchange 100 Index(FTSE 100), Standard & Poor’s 500 (S&P 500) and NYSE ARCA oil & gas Index (OilGas) with two different periods. In the Table 3 we show the results from the Gibbs sampler using data from year 2005 October to year 2010 December, while Table 4 shows the results for period between October 2010 and December 2015.

**Table 3:** Comparison between extracted parameters of different indices between year 2005 and 2010.

Parameters	DJI	S&P 500	NASDAQ 100	FTSE 100	OilGas
Drift	0.10245	0.11345	0.10625	0.11171	0.22821
Volatility	0.12574	0.14119	0.17082	0.14919	0.22557
Jump Frequency	19.33768	19.24939	14.06721	16.59051	15.91333
Jump Intensity	-0.00407	-0.00519	-0.00388	-0.00475	-0.01131
S.D of jump	0.04524	0.04711	0.05277	0.04554	0.06655

**Table 4:** Comparison between extracted parameters of different indices between year 2010 and 2015.

Parameters	DJI	S&P 500	NASDAQ 100	FTSE 100	OilGas
Drift	0.13043	0.14949	0.17544	0.06069	0.11527
Volatility	0.12365	0.12797	0.14913	0.14301	0.18886
Jump Frequency	5.23150	6.83121	6.07712	6.08239	12.74748
Jump Intensity	-0.00592	-0.00478	-0.00568	-0.00446	-0.00615
S.D of jump	0.05644	0.05294	0.05739	0.05095	0.04405

The Tables 3 and 4 show that the first period between 2005 and 2010 contains relatively more jumps compared to the second period between 2010 and 2015. The first period had a minimum frequency of 14 jumps across the indices. The intensity of jumps, however, had a smaller scale than expected, with an average value of 1%. We believe there are two reasons that caused the intensity of the jumps to be small. First, the jump parameter generates intermittent jumps, however, the net effects on these intermittent jumps are indistinguishable from the fluctuations generated from the volatility parameters. Secondly, the distribution of *Y*_*i*_ in Eq 2 is symmetric, hence occurrences of jumps can happen in both upward and downward directions. The cancelling effects of the upward jumps and downward jumps resulted in a small jump intensity value and with large standard deviation.

Comparing the values of the second period in Table 4 to the first period in Table 3, the values for jump intensity and its volatility do not differ by much, whilst the jump shows an obvious difference where most of the indices had an average frequency of jump of 6, besides NYSE ARCA OIL & GAS INDEX.

**Fig 2:**
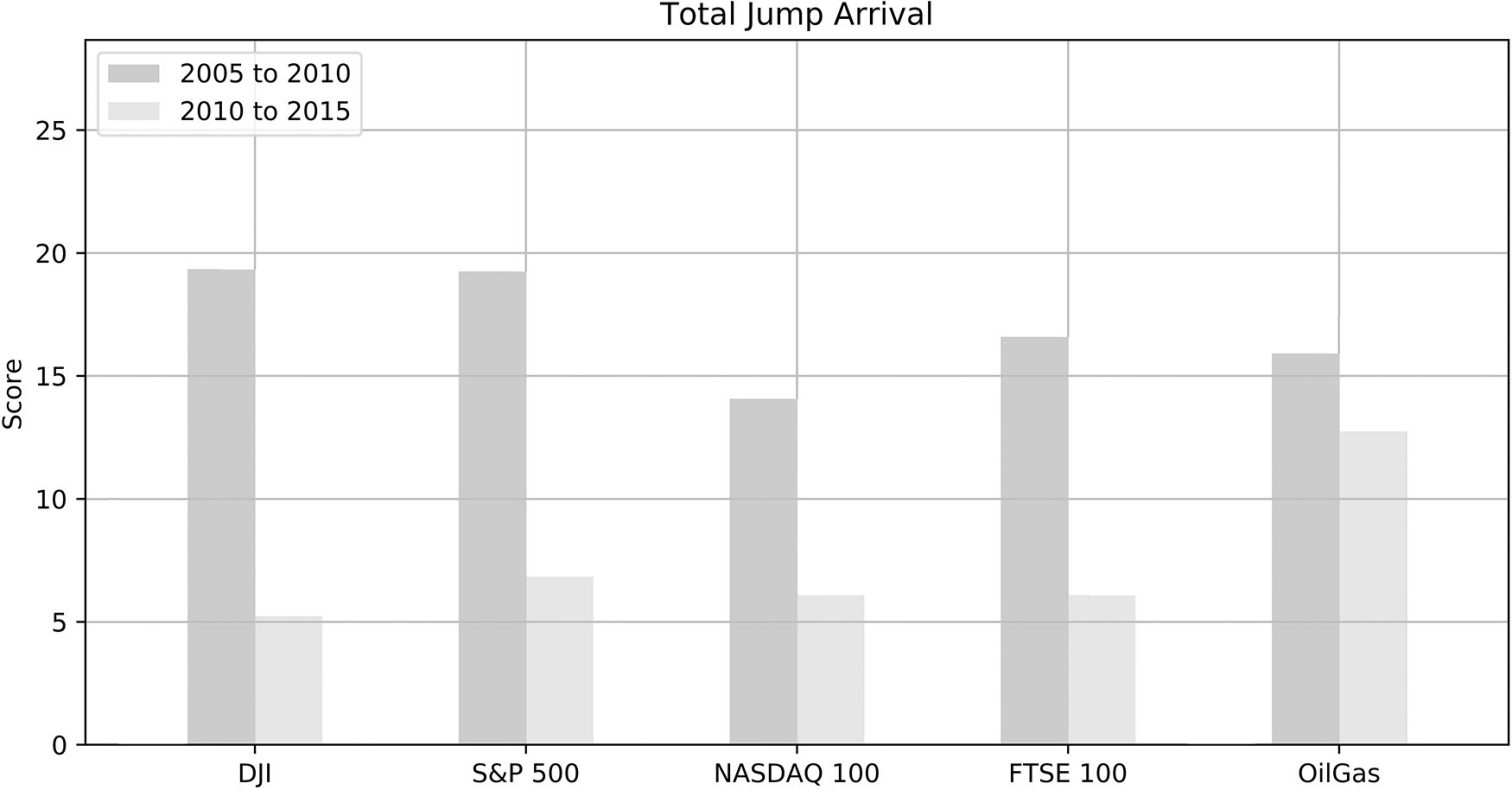
Jump frequency of different market indiecs for 2 different periods of times (2005 to 2010 and 2010 to 2015).

Fig 2 shows the frequency of jumps for each index from two different periods of time. We can see that the market had a high jump event between Oct 2005 to Dec 2010 as compared to the next 5 year period. There was a world economic recession during the year 2007 and 2008, the plummet of asset prices caused the number of jump arrivals to shoot up to nearly 20. Subsequently, the market was more stable after 2010; hence, the jump occurrences reduced to between five and six times a year.

The frequency of the jumps for NYSE ARCA Oil & Gas Index during the period of 2010 to 2015 was higher as compared to the other four indices. Although the average frequency of jumps had lowered to 6, the NYSE ARCA Oil & Gas index recorded a jump arrivals as high as 13; hence, we checked on the historical index prices throughout Oct 1, 2005, till Sept 30, 2015, as depicted in Fig 2.

**Fig 3:**
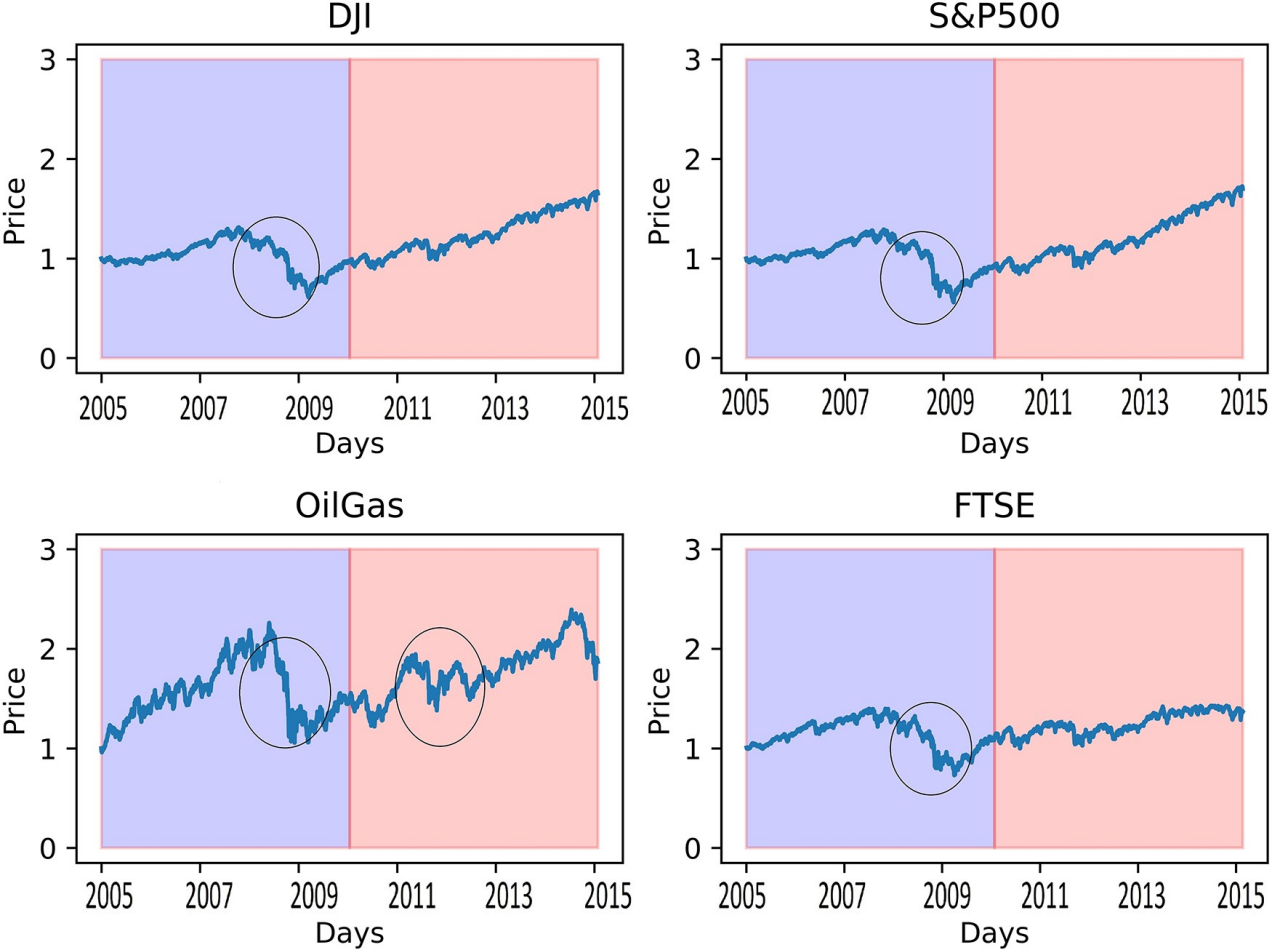
Comparison of price behavior for 4 indices. (Data retrieved from Yahoo Finance).

Fig 3 shows that there are some drastic changes between year 2008 to year 2009, and those regions are in the circles. We can see that the number of jumps of NYSE ARCA OIL & GAS INDEX remains at a higher level due to the sudden drop in prices in the second period (around 2012), while the other indices had started getting slightly more stable. Until year 2015, the oil and gas prices remain highly volatile from time to time, hence the frequency of jumps that remain at a higher level is reasonable. This shows that the Gibbs sampler could provide good estimation on the frequency of jumps that occurred over the time period; however it remains weak in estimating the jump intensity.

In the next section, we studied the asymmetric diffusion model with European call options and annuity and investigated its impact on the pricing of both instruments. This is to study if the jump model could retain its characteristics for two different types of instruments.

### Pricing European calls with asymmetric jump diffusion model

We would like to determine how much the impact of jumps had on the price of an European call option. Here, we had simulated a asymmetric jump diffusion model with the initial price, *S*_0_ of 100, with a strike price, *K* = 100. The drift *μ* is set to be a constant following the underlying asset, where it is -0.005 for DJI index. The volatility *σ* is constructed with Dupire’s formula under a risk neutral measure in order to reduce arbitrage opportunities [33, 34].

$$\begin{array}{c} \hat{\sigma }\left(T,K\right)=\frac{1}{K}\sqrt{\frac{2\delta C/\delta T(T,K)}{\delta^{2}C/\delta x^{2}\left(T,K\right)}} \end{array}$$ (11)

The constructed volatility of DJI index over 20 years is 0.00555, using Dupire’s formula.

The price of the European call option based on the GBM model is calculated using the Black-Scholes formula. On the other hand, the price of the call option based on the asymmetric jump diffusion model can also be calculated by simulating Eq 4 [35].

**Fig 4:**
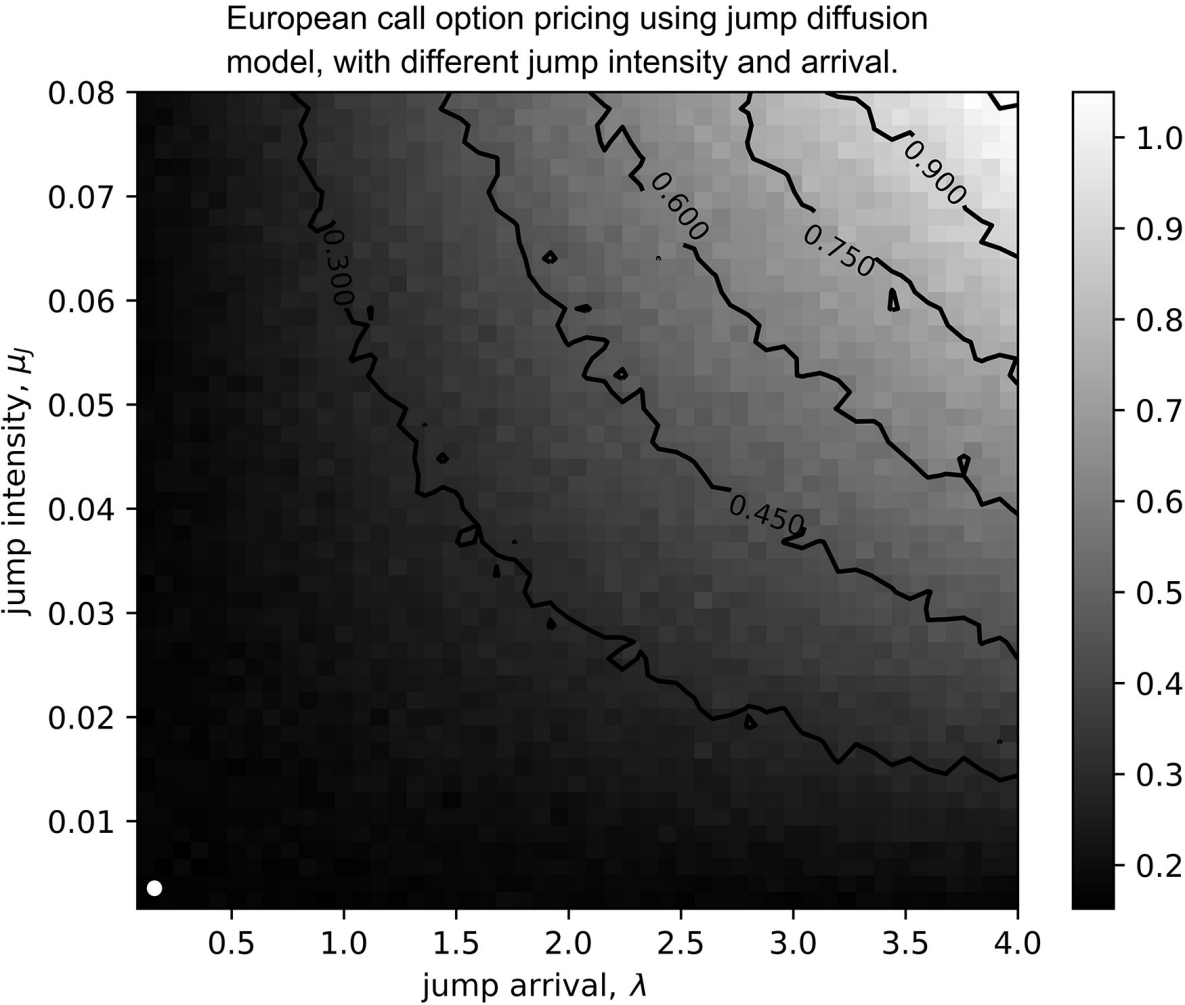
The expected call option price for different jump intensity and frequency.

Both results are mapped on to a contour with horizontal axis denoting the range of the frequency of the jumps and the vertical axis denoting the jump intensity. The frequency of the jumps, λ, ranges from zero (no jump event, i.e., the GBM model) to four (indicating four jumps per year), while the jump intensity ranges from 0 to 0.8. (0 < λ <4, 0 <*μ*_jump_ <0.08). The results are shown in Fig 4.

The white dot at the left bottom in Fig 4 is the payoff of the European call option with GBM. Along the vertical axis, when jump had zero arrival, we can observe that the call option’s payoff fluctuates around zero.

When the jump events occur, the payoff from the asymmetric model is greater than the expected payoff of the Brownian motion and increases proportionally to the increment of the jump intensity. From the result, as the intensity of jump increases, the payoff increases even higher. The highest peak occurs, when the jump intensity and frequency are at their highest levels.

The results in Fig 4 consider only the positive jumps. Although a large asset price downswing could occur upon negative jumps, a call option is always protected from the downside risk and hence no impact on the payoff.

In the following context, Dow Jones index and S&P 500 are calculated with the empirical method we mentioned previously. The results can be referred to in Table 2. The average drift of the Dow Jones index and the S&P 500 over 20 years from year 1995 to 2015 are -0.0050198 and -0.0052949 respectively, therefore we take the average value of -0.00515. The contructed volatility for both indices is 0.0055 using Eq 11.

We will use the data from the S&P 500 index and the Dow Jones index and compare them with the European call option. After we evaluated the Dow Jones index and the S & P 500, the jump frequency we get can range from 0 to 11 (during year 1996 in Table 2). The jump intensity will go up 0.0052 during year 2008; hence, we set value ranges of 0 to 12 and 0 to 0.05 for the frequency of jump and the intensity of jump, respectively (0 < λ <12, 0 <*μ*_jump_ <0.05). An event with 0 jump frequency is similar to the standard geometric Brownian motion model.

Using the market parameters with the value we mentioned aboved, we can construct the countour from the pricing model that uses the asymmetric jump model. The countour will show the value of the option, with different frequencies of jump and jump intensities.

**Fig 5:**
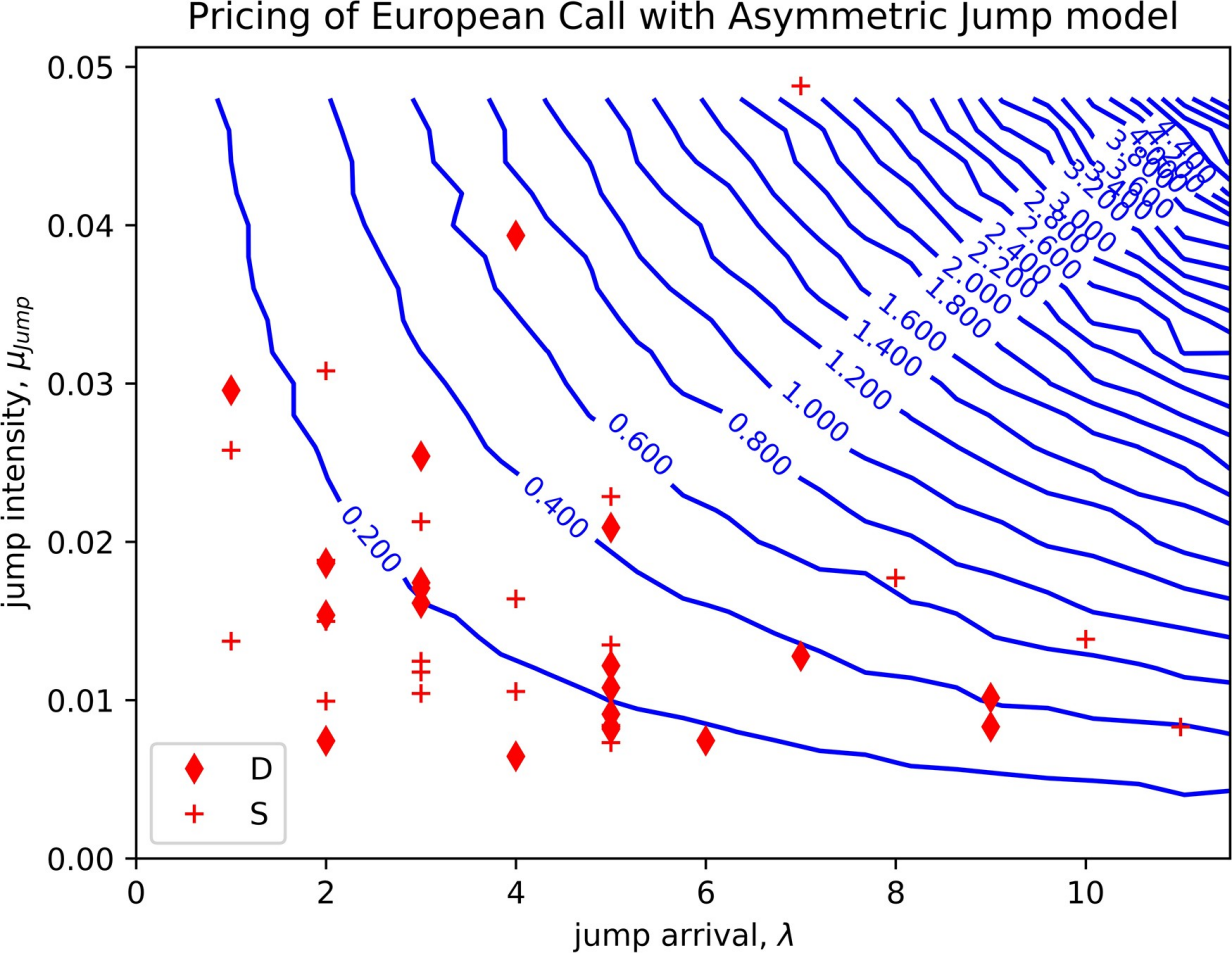
The contour of the expected payoff for 1-year at-the-money European call based on empirical asymmetric jump diffusion model. The parameters are estimated from 20 years of S&P 500 index (cross) and Dow Jones index (diamond).

From Fig 5, the diamond marks the estimated yearly positive jump frequencies and jump intensities for the Dow Jones (DJI) based on the data in the past 20 years from 1995 to 2015. The contour lines are the expected payoff of the European call based on the empirical asymmetric jump diffusion model. Fig 5 shows that the pricing from standard GBM is not good enough to compensate risk from volatile periods. We observe that the expected payoff would differ from the Black-Scholes formula and the difference can be as high as 0.7 from the normalized return.

Similarily, the cross sign in Fig 5 shows the expected positive jump frequency and jump intensities for the S&P 500 over the past 20 years from 1995 to 2015. The highest attainable option price using the S&P 500 could exceed 1.0 from the normalized return. This observation shows that, for different indics or assets, the impacts of the jump to the option prices can differ significantly.

### Annuity pricing with asymmetric jump diffusion model

We also investigated the impacts of the frequency of the jumps on the price of an annuity. We plotted the contour of the annuity’s price based on the Heston model in Eq 10 [30]. Similar to the case of the European call option, we set the value ranges of 0 to 12 and 0 to 0.05 for the frequency of jump and the intensity of jump, repectively (0 < λ <12, 0 <*μ*_jump_ <0.05). Zero jump frequency corresponds to the standard geometric Brownian motion model. Then, the sub-account value *A*_*t*_ was simulated based on Eq 8 and the price of the annuity was calculated.

**Fig 6:**
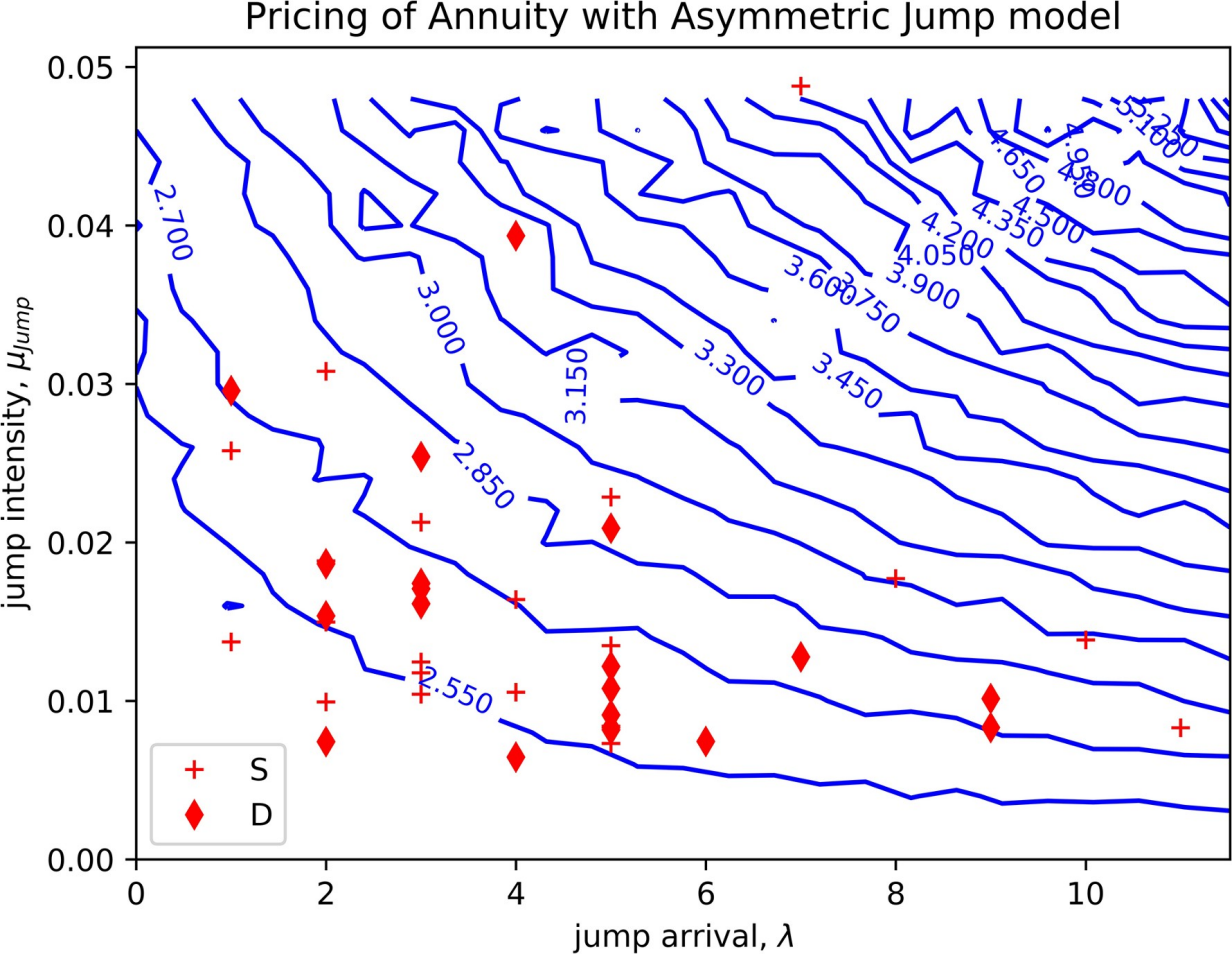
The contour of the value of an annuity based on empirical asymmetric jump diffusion model. The parameters are estimated from 20 years of S&P 500 index (cross) and Dow Jones index (diamond).

Fig 6 shows the contour of the calculated annuity price for different jump frequencies and intensities. The cross and diamond mark the jump frequencies and intensities for the S&P 500 index and the Dow Jones Index for the 20 years. The diamond mark shows the pricing payoff that is evaluated based on the Dow Jones index using data from year 1995 to year 2015. The cross mark was evaluated based on the S&P 500 index, and with the same year period as DJI.

In Fig 6, the price of an annuity corresponding to the GBM is located at the bottom left corner with a value that is less than 2.4. However, from the frequencies and intensities corresponding to the historical data, we can see that in some of the years the value of the annuity can go above 3.0. This shows that without considering the chances of positive extreme events, the annuity is undervalued. Vice versa, the expected payoff of pricing would suffer a higher loss upon a series of negative or downward jump events that cause the annuity policy to be overvalued. In either case, the estimated value of the annuity in our model differs from the value in the model that assumes no jump. This indicates that additional risk exposure for the pricing company and the empirical asymmetric jump diffusion model provides a better way to estimate this additional risk.

## Conclusion and discussion

Both economic downturn in 1998 and the subprime crisis in the year 2007 are the most noticeable extreme events that happened in the economy. It is undeniable that extreme events do exist in the market or even single asset. The ability to capture its signal before it happens is significant and important so that it can help reduce the risk borne by investors.

The jump diffusion model is definitely more useful in capturing such signals than observing the past prices or market behavior such as moving average and trend. As for the Black-Scholes model, we had shown that the inability to capture the extreme jump event would lead the investor into a riskier situation that the BS model could not foresee.

This research had identified the effect of jump on European call options with different jump intensities and frequencies. The results show that the drift of jump, *μ*_jump_ and frequency of jump, λ will affect the option payoff. The result will be either higher or lower depending on the drift of jump. Even the drift is zero, the payoff would be affected too, as the volatility of jump would alter the prices. This means that, whenever we expect a jump from the stock or market, even if the drift is small, the risk of volatility is still existing. As long as there is a jump, there will be larger risk and a fluctuation of price should be expected.

The Gibbs sampling technique could provide the values and parameters of the market data, which is the drift and volatility of the model itself, the frequency of jumps and the drift and volatility of the jump. However in the context of the jump diffusion model, it cannot converge everytime. Sometimes, the same path and pattern of a stock could be simulated using different sets of market parameters. For example, a high drift low volatility stocks path might be able to be attained by a slightly lower drift but higher volatility. Hence, the results attained had a range of possibilities.

However, if the frequency of jumps and jump intensity is high, the stock itself is undergoing several jump events and a huge fluctuation in price is expected in the near future.

The jump diffusion model does not solely fit in options only. It could help in calculating the expectations of other derivatives. The payoff of annuity is shown in results that, the reward function could be higher if the underlying assets are undergoing a positive jump event. Similarly, there is a large risk of loss if it is going to undergo a negative jump event. In this case, we can say that the annuity is undervalued, as the risk being undertaken is far larger than what is expected. For example, we refer to Fig 6 from the previous section, the price should be somewhere around 2.6 rather than 2.3 without considering the risk of jump.

There could be other derivatives and purposes that the jump diffusion model can fit into it for better risk managing and loss preventing. Hence, it is important to consider jump model than geometric Brownian motion when dealing with market derivatives.
